# DR2 blocker thioridazine: A promising drug for ovarian cancer therapy

**DOI:** 10.3892/ol.2020.11285

**Published:** 2020-01-09

**Authors:** Min Yong, Tinghe Yu, Si Tian, Shuaibin Liu, Jiao Xu, Jianguo Hu, Lina Hu

Oncol Lett 14: 8171-8177, 2017; DOI: 10.3892/ol.2017.7184

Subsequently to the publication of the above article, the authors have realized that Figs. 1 and 2 contained incorrect GAPDH control bands; in particular, the same GADPH bands had erroneously been included twice in Fig. 2 for experiments that involved two different cell lines. These GAPDH bands were included inadertently in this pair of figures owing to a mislabelling of the original data files.

The corrected versions of Figs. 1 and 2, as they should have appeared in the Journal, are shown opposite. All the authors agree to this Corrigendum. Note that the revisions made to these figures do not adversely affect the results reported in the paper, or the conclusions stated therein. The authors regret that these errors were not noticed prior to the publication of this article, and offer their apologies to the Editor and to the readers of the Journal.

## Figures and Tables

**Figure 1.**
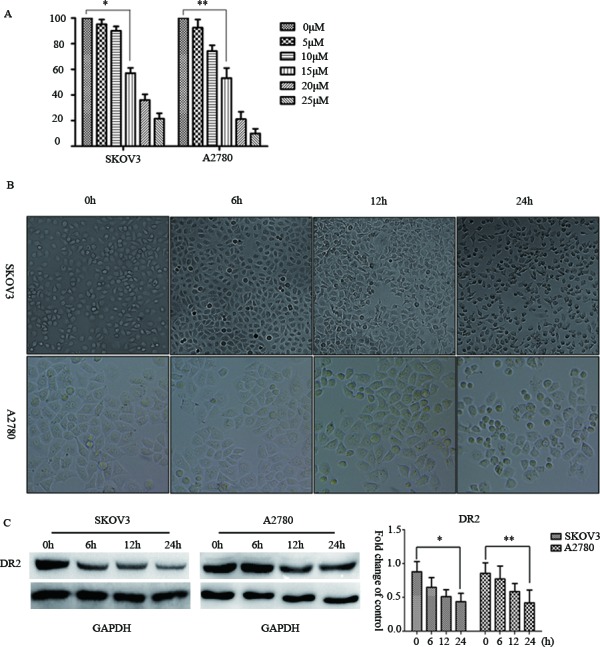
Thioridazine blocked DR2 and suppressed cell proliferation. (A) The effect of thioridazine on cell proliferation; a concentration of 15 µM was the minimum required to induce the inhibition of SKOV3 and A2780 cells. Data are presented as the mean ± standard derivation of three independent experiments. *P=0.003; **P=0.009 vs. control. (B) Light microscopy images of ovarian cancer cell morphology following treatment with 15 µM thioridazine for the designated length of time (0, 6, 12 and 24 h). Scale bar, 100 µm. (C) DR2 expression as determined by western blotting analysis. GAPDH was used as the reference. Bar graph represents the mean ± standard deviation of triplicate experiments. *P=0.019, **P=0.038, compared with the control group.

**Figure 2.**
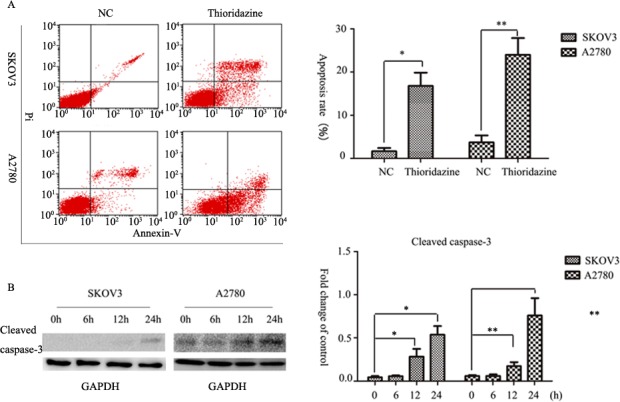
Thioridazine-induced apoptosis. (A) Annexin V/PI double staining analysis of SKOV3 and A2780 cells treated with or without 15 µM thioridazine. Data are representative of the results of triplicate independent experiments. *P=0.01, **P=0.005, compared with the control group. (B) Representative western blotting demonstrating the levels of cleaved caspase-3. GAPDH was used as an internal control. Bar graph represents the mean ± standard deviation of triplicate experiments. *P=0.041, 12 h; **P=0.012, 24 h; ***P=0.044, 12 h; ****P=0.026, 24 h, vs. control group. PI, protease inhibitor; NC, negative control.

